# Reconstructing mutational lineages in breast cancer by multi-patient-targeted single-cell DNA sequencing

**DOI:** 10.1016/j.xgen.2022.100215

**Published:** 2022-11-09

**Authors:** Jake Leighton, Min Hu, Emi Sei, Funda Meric-Bernstam, Nicholas E. Navin

**Affiliations:** 1 Department of Genetics, UT MD Anderson Cancer Center, Houston, TX 77030, USA; 2 Department of Systems Biology, UT MD Anderson Cancer Center, Houston, TX 77030, USA; 3 Graduate School of Biological Sciences, UT MD Anderson Cancer Center, Houston, TX 77030, USA; 4 Department of Precision Oncology, UT MD Anderson Cancer Center, Houston, TX 77030, USA; 5 Department of Bioinformatics and Computational Biology, UT MD Anderson Cancer Center, Houston, TX 77030, USA

**Keywords:** single-cell genomics, triple-negative breast cancer, intratumor heterogeneity, mutational evolution, breast cancer

## Abstract

Single-cell DNA sequencing (scDNA-seq) methods are powerful tools for profiling mutations in cancer cells; however, most genomic regions sequenced in single cells are non-informative. To overcome this issue, we developed a multi-patient-targeted (MPT) scDNA-seq method. MPT involves first performing bulk exome sequencing across a cohort of cancer patients to identify somatic mutations, which are then pooled together to develop a single custom targeted panel for high-throughput scDNA-seq using a microfluidics platform. We applied MPT to profile 330 mutations across 23,500 cells from 5 patients with triple negative-breast cancer (TNBC), which showed that 3 tumors were monoclonal and 2 tumors were polyclonal. From these data, we reconstructed mutational lineages and identified early mutational and copy-number events, including early *TP53* mutations that occurred in all five patients. Collectively, our data suggest that MPT can overcome a major technical obstacle for studying tumor evolution using scDNA-seq by profiling information-rich mutation sites.

## Highlights

• Developed a custom targeted panel to profile information-rich mutations via scDNA-seq
• Pooled mutations profiled by bulk exome-seq from 5 triple-negative patients
• Applied MPT to 330 mutations across 23,500 cells using microfluidics-based scDNA-seq
• Reconstructed mutational lineages and CNA events underlying breast cancer evolution

## Graphical abstract

**Figure:**
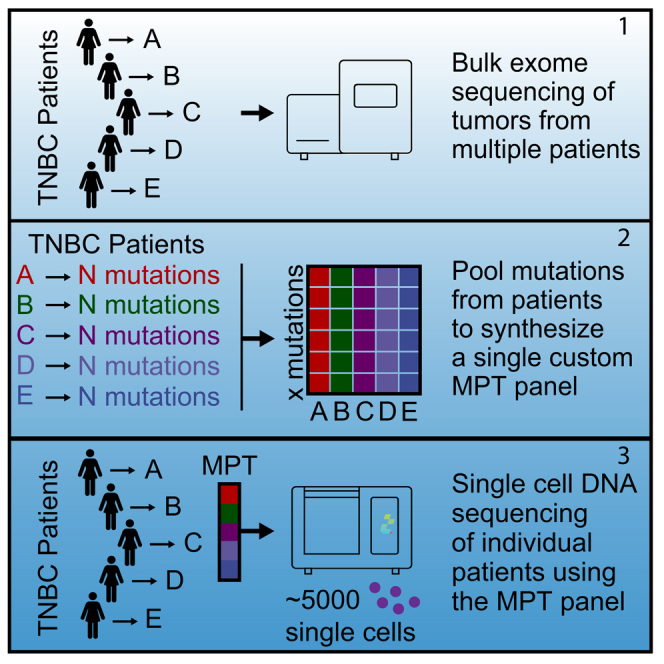

## Teaser

Leighton et al. developed a multi-patient-targeted (MPT) panel to profile information-rich mutation sites in single cancer cells by synthesizing a custom targeted panel using pooled mutations from patients with cancer who were first profiled by bulk exome sequencing. Along with high-throughput scDNA-seq on a microfluidics platform, MPT was applied to profile 330 mutations across 23,500 cells from 5 patients with triple-negative breast cancer. This reconstructed mutational lineages and copy-number events, including early *TP53* mutations observed in all five patients, underlying breast cancer evolution.

## Introduction

Triple-negative breast cancer (TNBCs) is an aggressive subtype that is characterized by a lack of estrogen receptor (ER), progesterone receptor (PR), and Her2 receptor levels. Patients with TNBC frequently develop resistance to chemotherapy (∼50%) and progress to metastatic disease, often leading to poor survival.^1^^,^^2^^,^^3^ In contrast to ER-positive breast cancer, patients with TNBC display extensive copy-number alterations (CNAs) and driver mutations in *TP53* in 83% of patients.^4^ Furthermore, patients with TNBC have been shown by single-cell DNA sequencing (scDNA-seq), multi-region sequencing, and whole-genome sequencing methods to display extensive intratumor heterogeneity (ITH).^5^^,^^6^^,^^7^ This ITH may explain why patients with TNBC frequently develop resistance to chemotherapy and often progress to metastatic disease.^8^^,^^9^ Previous studies have shown that TNBCs evolve through punctuated copy-number evolution (PCNE), in which large numbers of CNA events are acquired in short bursts of evolution at the earliest stages of progression.^3^^,^^5^^,^^10^^,^^11^ However, the dynamics and timing of mutations during TNBC progression represent a fundamental gap in knowledge. Additionally, improved knowledge of ITH is important for the clinical diagnosis and treatment of patients with TNBC, particularly when selecting targeted therapies. One approach for resolving intratumor heterogeneity that is gaining broader use in both research and clinical studies is scDNA-seq.^12^

The field of single-cell genomics has shown remarkable progress in the development of both transcriptomic and genomic profiling methods over the last 10 years.^13^ Initial scDNA-seq for profiling the genomes and exomes of single cells were low throughput and had extensive technical errors.^14^^,^^15^^,^^16^ These technologies have been improved through developments in whole-genome amplification (WGA) chemistries^15^^,^^17^^,^^18^^,^^19^ and the implementation of nanowell and microfluidic platforms.^20^^,^^21^^,^^22^^,^^23^^,^^24^ Specifically, single-cell genomic copy-number profiling technologies have undergone substantial improvements with the use of tagmentation chemistries and microfluidic or nanowell platforms (10X Genomics CNV, ACT, DLP+).^11^^,^^25^ Similarly, scDNA-seq platforms for mutational profiling have been developed using microfluidic platforms (Mission Bio, Tapestri), which have enabled the profiling of tens of thousands of cells in parallel.^26^^,^^27^^,^^28^^,^^29^^,^^30^^,^^31^ However, in contrast to genomic copy-number profiling, which involves unbiased whole-genome sequencing (WGS) at sparse depth, mutational profiling requires high-coverage data. Consequently, it is necessary to target genomic regions for sequencing (e.g., exome, cancer gene panels) in order to make these assays economically feasible. This presents a major challenge since the informative mutation sites are not usually known *a priori*, and thus most of the genomic regions profiled in single cells contain only reference DNA sequences or germline SNPs.

To address this challenge, we developed a multi-patient-targeted (MPT) scDNA-seq sequencing approach that combines bulk DNA sequencing, single-cell droplet-based microfluidics, and a custom targeted panel. We applied MPT to 5 invasive TNBC tumor samples, which resolved the clonal substructure of the tumors and enabled the reconstruction of clonal lineages during tumor progression. Our data show that *TP53* mutations and other early mutational events in TNBC progression are accompanied by CNA events, leading to the expansion of the primary tumor mass. Additionally, our data show that most mutations are early events in the progression of TNBC tumors and that subclones undergo stable clonal expansions with only limited intermediate mutations that are acquired during tumor growth.

## Results

### MPT panel sequencing

**Figure 1:**
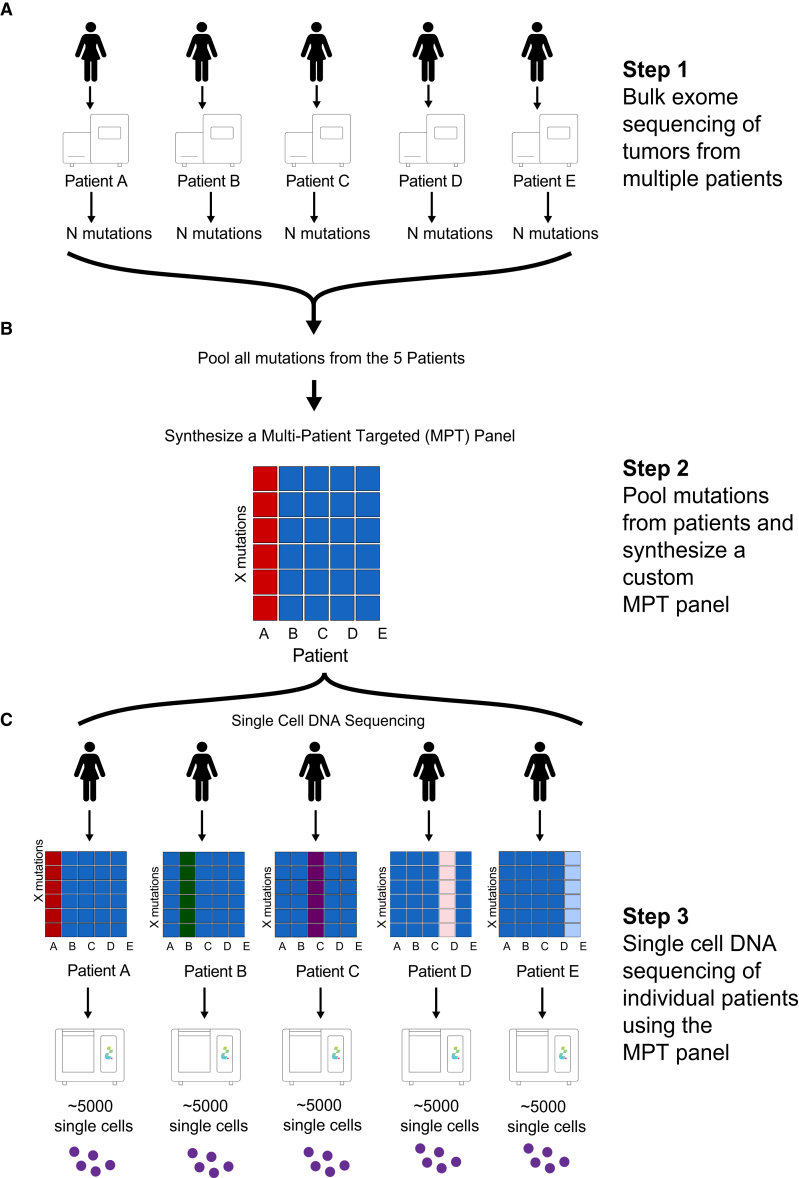
Overview of multi-patient-targeted single-cell sequencing workflow (A) Bulk exome sequencing is performed on a cohort of human tumors. (B) Mutations from all patients are pooled together and used to synthesize a multi-patient-targeted (MPT) custom panel. (C) The MPT panel is used to perform single-cell DNA sequencing (scDNA-seq) on each patient individually to profile thousands of cells in parallel using a microfluidics platform.

A major challenge with targeted scDNA-seq of mutations in tumors is that generally only a few somatic mutations (e.g., 1–10) can be profiled from each patient using an unbiased panel that covers a small region (e.g., 5 Mb) of the human genome, while most genomic regions sequenced contain only the reference genome bases and SNPs. This problem results in high costs for scDNA-seq of regions with limited useful mutational information and prohibits large numbers of cell to be profiled in parallel. To address this problem, we developed a method called MPT sequencing (Figure 1). In this approach, we first profile a set of patients (e.g., 5–20) with bulk deep-exome sequencing methods (approximately ∼200× tumor, ∼60× normal) to identify somatic mutations and then pool all mutations together from the patient cohort (e.g., ∼400 mutations) to develop a custom targeted panel for scDNA-seq that targets all of the mutation sites across all of the patients (Figure 1A). In future developments of the microdroplet platform (Mission Bio), larger amplicon panels (e.g., thousands of targets) will allow more patients to be profiled or a larger number of mutation sites to be profiled per patient.

The custom panel is synthesized commercially (V1 custom targeted panel, Mission Bio) for the purpose of downstream scDNA-seq using a microfluidics platform (Tapestri, Mission Bio) (Figure 1B). We then perform scDNA-seq (Mission Bio) using the same MPT panel on each of the tumors that were previously analyzed by bulk exome sequencing to profile ∼5,000 cells for each tumor (Figure 1C). In the scDNA-seq experiments that are performed for each tumor, the mutations are profiled for each patient (e.g., ∼50 mutations), as well as the reference sites for the mutations in the other patients (which are not utilized in the downstream analysis). The major advantage of the MPT approach is that it greatly mitigates the sequencing space for non-informative genome information that would normally be profiled using an unbiased panel. Notably, when the same mutations occur in different patients, these sites will save additional space on the custom MPT panel. By focusing the scDNA-seq on the mutation sites of interest, the sequencing costs are greatly decreased, enabling very high-throughput analysis of thousands of single cells in parallel at low costs for each patient.

### Bulk exome sequencing of TNBCs

**Figure 2:**
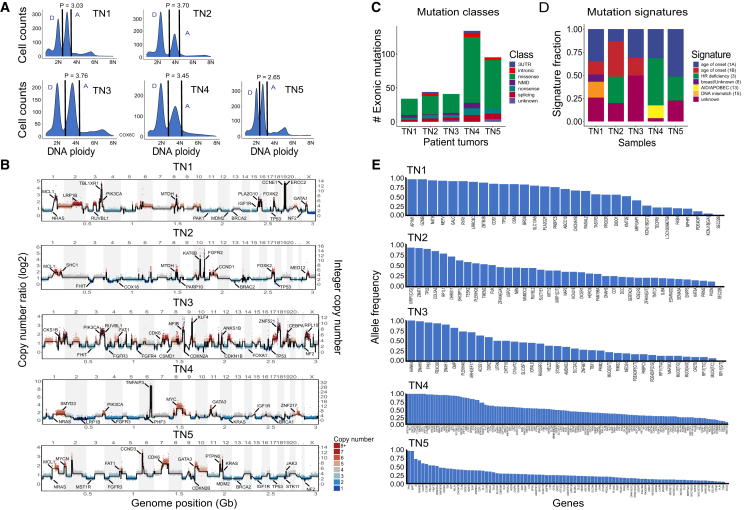
Bulk exome sequencing and mutational analysis of TNBC tumors (A) Frozen tumor tissues were dissociated into nuclear suspensions and stained with DAPI for FACS to isolate aneuploid single cells by differences in DNA ploidy, where P indicates the mean tumor ploidy and D and A indicate diploid or aneuploid distributions, respectively. (B) Pseudo-bulk copy-number ratio plots generated from single-cell WGS data from each tumor sample with integer copy-number states calculated. (C) Classes of exonic mutations for each tumor identified by Annovar. (D) Mutational signatures calculated from the exonic mutations. (E) Allelic frequencies for all of the somatic mutations identified in each TNBC tumor that were used to design the MPT panel.

We selected frozen invasive tumor samples (ductal carcinoma *in situ* [DCIS]/invasive ductal carcinoma [IDC]) from 5 untreated patients with TNBC that were negative for the ER, PR, and Her2 receptor levels (evaluated by immunohistochemistry) and Her2 amplification (evaluated by cytogenetic analysis) for bulk exome sequencing followed by MPT profiling using a microfluidics system (Tapestri, Mission Bio) (Table S1). To enrich the bulk tumor cell populations, we first generated single nucleus suspensions and stained the nuclei with DAPI to perform fluorescence-activated cell sorting (FACS) (Figure 2A). Using FACS, we gated distributions of cells from 2.65–3.7(N) ploidy to enrich aneuploid tumor cell fractions. Using the aneuploid cells, we generated sequencing libraries and performed both WGS at sparse depth to estimate genomic copy number and exome capture (Roche, Nimblegen v.2) to identify point mutations (Table S2). The genomic copy-number profiles showed extensive aneuploidy and copy-number aberrations (CNAs) in all of the tumors, including amplifications of oncogenes such as *MYC* and deletions of tumor suppressors such as *TP53* (Figure 2B). Exome sequencing of the aneuploid-sorted nuclei and matched normal tissues identified a range of somatic mutations (mean = 66) in the tumors, in which TN4 and TN5 showed a highly elevated mutation burden compared with TN1–TN3, suggesting that they are hypermutators. Within the different classes of exonic mutations, missense mutations were the most prevalent (>70%) in all 5 samples, with smaller numbers of non-sense and splicing mutations identified (Figure 2C).

Analysis of the mutational signatures showed that several common breast cancer signatures were prevalent, including signatures 1a/b, 3, 13, and 15 (Figure 2D). Signature 1a/1b is aging (deamination of methylcytosines), signature 3 is homologous recombination repair deficiency, signature 13 is APOBEC cytidine deaminases, and signature 15 represents DNA mismatch repair. Interestingly, the highest mutation frequency in the cohort observed in TN4 correlates with the AID/APOBEC signature, suggesting a possible mechanism for the acquisition of the mutations. Finally, we investigated the distribution of somatic mutation frequencies, which ranged from ∼0.1% to ∼99% for each patient, and showed that *TP53* was one of the highest-frequency mutations identified in all 5 patients (Figure 2E). We combined all of the somatic mutations identified across the 5 patients with TNBC to design a custom MPT panel (Mission Bio) for scDNA-seq analysis.

### Mutational substructure of TNBC tumors

To resolve the clonal substructure of each TNBC tumor, we applied the MPT panel to perform scDNA-seq of a total of 23,526 cells (range: 4,002–5,941) of the 5 tumors using the microdroplet (Mission Bio) scDNA-seq platform (Table S3). In contrast to bulk sequencing, the scDNA Mission Bio experiments were all performed on unsorted cells (not subjected to FACS). The resulting data showed that 330 targeted sites (STAR Methods; Table S3) had 164× coverage depth across the 5 tumors. The somatic mutations were detected in each single cell by *de novo* variant calling using GATK, independently of the exome bulk data that also used GATK for variant calling (STAR Methods).

**Figure 3:**
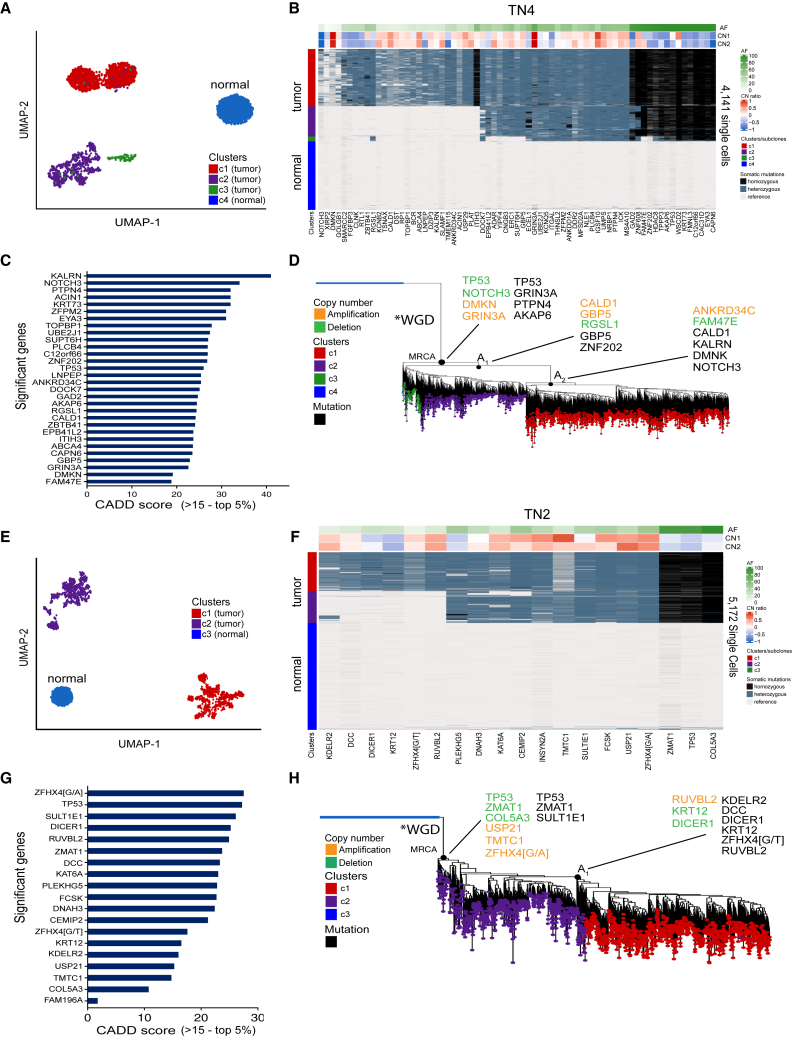
Clonal substructure and phylogenetic reconstruction of two polyclonal tumors (A and E) High-dimensional clustering of somatic mutations using UMAP of 4,141 and 5,172 (TN4, TN2) single cells to identify major clusters of subclones. (B and F) Heatmaps of hierarchical clustered mutations in TN4 and TN2 showing the clonal substructure and heterogeneity within the tumor subpopulations, with cluster-level copy-number estimations shown in the header tracks above. (C and G) CADD scores are used to rank functional impact of mutations’ deleteriousness in TN4 and TN2 (CADD >15, top 5%). (D and H) Phylogenetic reconstruction of the mutation lineages in TN4 and TN2, chronology, and timing of copy-number aberrations relative to the MRCA using a neighbor-joining tree. The common ancestors (A1, A2) are annotated on the tree, as well as the whole-genome doubling (WGD) events.

In the polyclonal tumor TN4, clustering of 4,141 single cells across 69 genes identified 4 major clusters in high-dimensional space using uniform manifold approximation and projection (UMAP) that corresponded to three tumor subclones (c1–c3) and one population of diploid cells (c4) (Figure 3A). A clustered heatmap of the mutations further revealed the somatic mutations in each subclone, including shared mutations that were present in all three tumor clusters (Figure 3B). This analysis identified 15 homozygous mutations that were shared among all three subclones, including *TP53*, which is the most commonly mutated gene in TNBC.^4^ The copy number of each gene on the targeted panel was estimated from the read depth, which identified chromosomal gains and losses (STAR Methods). These data showed that *GRIN3A*, *DMKN*, and *IGSF10* were amplified, while *NOTCH3* and 15 other genes with homozygous mutations showed copy-number losses. Computational prediction of the functional impact scores using CADD identified *KALRN*, *NOTCH3*, and *PTPN4* as having the highest functional impact scores, among other genes (Figure 3C).^32^

To infer the chronology and order of single-nucleotide variants (SNVs) and CNAs during tumor evolution, a neighbor-joining (NJ) tree was constructed from the mutation matrix, after which the mutations and CNA events were annotated (STAR Methods). The NJ tree showed three major branching points: the most recent common ancestor (MRCA), the first subclonal ancestor (A1), and the second subclonal ancestor (A2), which resulted in the divergence of the three tumor subclones (c1–c3). Truncal mutations that occurred in all tumor subclones included chromosome losses in *TP53* and *NOTCH3* and gains in DMKN and *GRIN3A*, while early mutations included *TP53*, *GRIN3A*, *PTPN4*, and *AKAP6*. The c3 subclone was closest to the MRCA, suggesting that it was one of the earliest subclones that diverged, consistent with the clustering results, which showed that it harbored the lowest number of mutations (n = 33). The c2 clone diverged after the A1 ancestor by acquiring an additional set of mutations (n = 8). Finally, the c3 clone diverged from the A2 ancestor via the acquisition of a large set of somatic mutations (n = 28) that included a mutation in *NOTCH3* (Figure 3D). Interestingly, a lack of gradual intermediate mutations was identified between the 3 major subclones in this analysis, and most mutations were very clonal within the three tumor subpopulations, which shared a common evolutionary lineage.

In the polyclonal tumor TN2, a total of 5,172 single cells were sequenced, and 19 somatic mutations were identified. Clustering of these data in high-dimensional space identified 3 major clusters, including two major tumor subclones (c1–c2) and one diploid cell cluster (c3) (Figure 3E). Hierarchical clustering of the somatic mutations identified 13 mutations that were shared between the two tumor clusters including 3 homozygous mutations, of which one was a *TP53* mutation (Figure 3F). The clustered heatmap also showed 6 mutations that were exclusive to the c2 subclone, suggesting that this subpopulation continued to evolve additional mutations after a shared common ancestor. Applying targeted copy-number inference from the read depth data, we identified amplifications in *ZFHX4*[G/A], *USP21*, and *TMTC1*, while *TP53*, *ZMAT1*, and *COL5A3* showed copy-number losses and had homozygous mutations (Figure 3F). CADD predictions of the functional impact scores showed that *ZFHX4*[G/A], *TP53*, *SULT1E1*, and *DICER1* had the highest scores in TN2 (Figure 3G). An NJ tree was constructed and showed two major branching points in evolution: the MRCA and A1. Early truncal events that occurred prior to the MRCA included chromosome losses in *TP53*, *ZMAT1*, and *COL5A3*, while gains included *USP21*, *TMTC1*, and *ZFHX4*[G/A] and mutations included *TP53*, *ZMAT1*, and *SULT1E1* (Figure 3H). The c1 clone diverged from c2 by acquiring 6 additional mutations including *DCC*, *DICER1*, and *RUVBL2* with high functional impact scores. Consistent with TN4, the mutational substructure of TN2 showed a lack of gradual mutations detected between the two subclones and the diploid cells.

**Figure 4:**
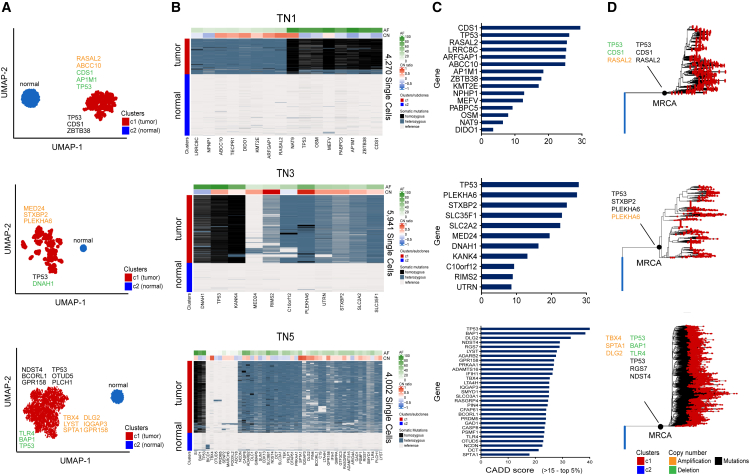
Clonal diversity and mutational lineages of three monoclonal tumors (A) High-dimensional clustering using UMAP of 4,270 (TN1), 5,941 (TN3), and 4,002 (TN5) single cells, respectively, identified one major tumor cluster and one cluster of normal cells in each sample. (B) Hierarchical clustering heatmaps of the mutations in TN1, TN3, and TN5 showing a monoclonal population of tumor cells, with cluster-level copy-number estimations for each mutation shown in the header bar. (C) CADD score is used to rank mutations’ predicted deleteriousness impact (CADD >15, top 5%) in TN1, TN3, and TN5. (D) Phylogenetic reconstruction of the TN1, TN3, and TN5 mutation lineages, chronology, and timing of copy-number aberrations relative to MRCA using a neighbor-joining tree with the MRCA annotated.

Clustering of TN1, TN3, and TN5 revealed mono-clonal tumors that were comprised of a single population of tumor cells and a single population of diploid cells (Figure 4). In TN1, 16 mutations were detected across 4,270 single cells. In TN3, the data identified 11 mutations across 5,941 cells, while in TN5, 47 mutations were detected across 4,002 cells (Figures 4A and 4B). In TN1, the mutations with the highest impact scores included homozygous mutations in *TP53*, *CDS1*, *RASAL2*, and *ARFGAP1*, while in TN3, these mutations included *TP53*, *PLEKHA6*, *STXBP2*, and *MED24*. In TN5, significant mutations were identified in *TP53*, *RGS7*, and *NDST4*, among other genes (Figure 4C). The CADD analysis showed that homozygous mutations in *TP53* had the most significant functional impact scores in all three tumors (Figure 4C). While most mutations in the three monoclonal tumors were detected in all tumor cells, there were a few mutations identified that occurred at lower frequencies, including *MED24* (4.8%) in TN3 and *OTUD5* (3.2%) and *PRDM5* (2%) in TN5, as well as *SENP6*, *MARCH6*, *PODXL2*, and *ADCY8* (all at 1.8%) of cells in TN5, suggesting that these were later lineage events that emerged during tumor progression.

Targeted copy-number inference showed that homozygous mutations *TP53*, *CDS1*, *AP1M1*, *ZBTB38*, *PABPC5*, and *OSM* were deleted in TN1, while *PLEKHA6* was amplified in TN3 and *TBX4*, *DLG2*, and *SPTA1* were amplified in TN5 (Figure 4B). NJ trees were constructed to infer the chronology and order of SNVs and CNAs, which showed that most events were truncal and occurred prior to the MRCA, after which the tumor cells expanded to form the tumor mass in each patient (Figure 4D).

### Comparison of clonal substructure from single-cell and bulk data

**Figure 5:**
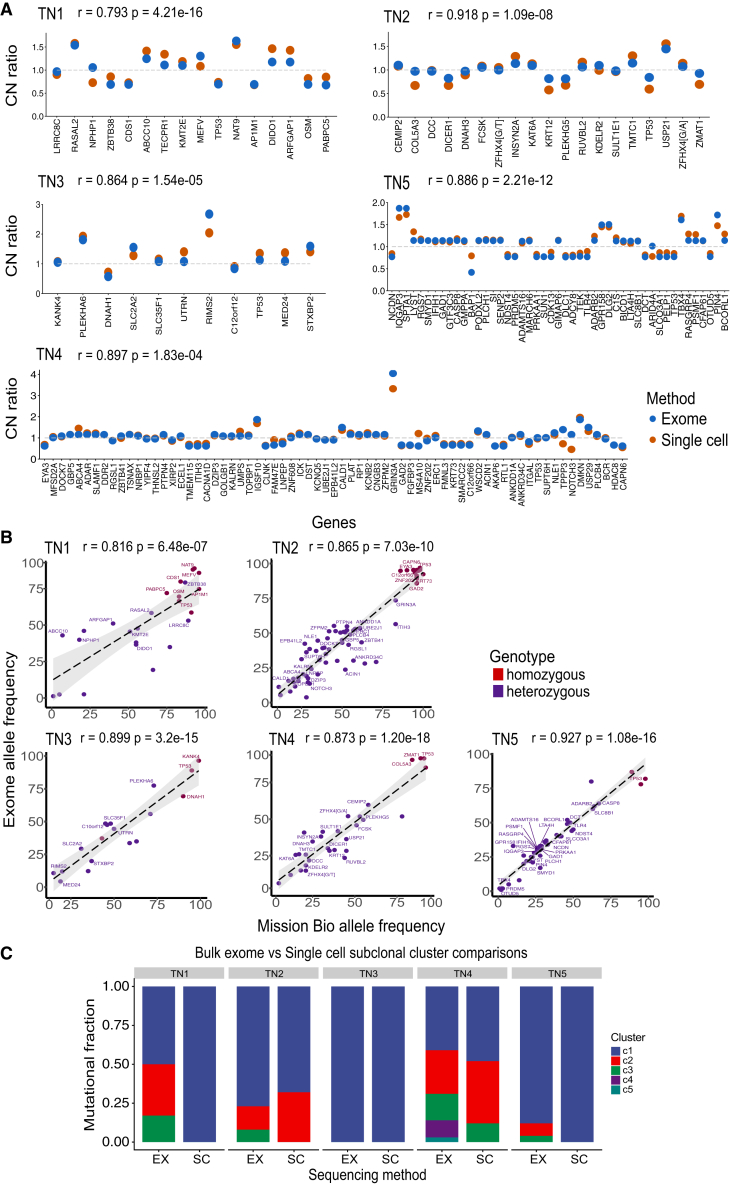
Comparison of MPT single-cell versus bulk exome sequencing data (A) Copy-number states for each gene were estimated across all tumor cells from each patient using the Mission Bio data and compared with a pseudo-bulk single-cell consensus copy-number reference from WGS data (Pearson correlation). (B) Average allele frequencies for each mutation calculated across all single cells from each patient in the MPT data are compared with the bulk exome reference (Pearson correlation). (C) Pyclone2 subclone clustering of mutation frequencies from bulk exome data compared with subclone clusters detected from MPT scDNA-seq profiling in each patient.

To understand the advantages of the MPT method for resolving clonal substructure, we directly compared data as “pseudo-bulk” with the single-cell MPT data in 5 patients. To assess the accuracy of the copy-number data, we first calculated a consensus of the single-cell MPT data and compared it with the pseudo-bulk single-cell WGS data, which we considered the “gold standard” reference for each patient (Figure 5A). Calculation of the Pearson correlation coefficients showed high correlation values (mean = 0.871) across the patients, suggesting that the single-cell MPT read count data, when merged together across cells, accurately reflected the pseudo-bulk WGS copy-number profiles. Notably, all high-level amplifications were detected in the single-cell data from TN1 (*RASAL2*, *NAT9*), TN2 (*USP21*), TN3 (*RIMS2*), TN4 (*GRIN3A*), and TN5 (*IQGAP3*, *SPTA1*, *TBX4*, *PIN4*). Next, we compared the variant allele frequencies (VAFs) of the bulk exome data versus the combined single-cell mutation frequencies (Figure 5B). Calculation of the Pearson correlation coefficients of the VAFs between the two datasets showed a high correlation (mean = 0.876) across the 5 patients, suggesting that they were highly concordant.

Finally, we investigated whether the clonal composition of the bulk exome data could be estimated by clustering VAF distributions using PyClone2 (Figures 5C and S4).^33^ These data showed that PyClone2 often overestimated the number of subclones present in each tumor, in which the two monoclonal TNBC tumors (TN1, TN2) were estimated to have 3 subclones by PyClone2 and one monoclonal tumor (TN4) was shown to have 1 subclone. For the two polyclonal tumors, Pyclone2 estimated a higher number of subclones in the exome data of TN3 (3 by single cell, 5 by exome) and in TN5 (2 by single cell and 3 by exome). These data highlight the challenge in estimating the number of subclones from bulk exome data based on VAFs, which single-cell mutation data can accurately resolve.

### MPT benchmarking metrics

To benchmark the sensitivity and accuracy of the MPT approach, we first investigated the variance of coverage by calculating the Gini index (GI) and coefficient of variation (CV) from the coverage read depths across all amplicons and cells (Figures S1A, S1B, and S2B). The average GI for amplicons was 0.32, while the average GI for cells was slightly higher at 0.51. Similarly, the CV for the amplicons was 65.6, and the average CV was 125.3. Additionally, we calculated the GI for each respective amplicon to understand the target-specific variation in coverage depth (Figure S2A). These data show that the coverage depth was higher than 100× coverage for most targeted sites and showed substantial variation that corresponded to the targeted genomic region, reflecting differences in amplification efficiencies across the genome (Figure S2B). Next, we calculated the average allelic dropout percentage (∼9%), the average doublet frequency (∼8%) across all samples, and the percentage of successful amplicons (∼85%), which showed good performance metrics for the single-cell datasets (Figures S1C–S1E and S5).

Next, we tested the robustness of the MPT approach by downsampling the FASTQ files of each tumor sample from 100× to 10× coverage (Figures S1F and S1G). This analysis showed that as the coverage depth decreased, most mutations in amplicons were still detected until ∼60× coverage depth, where a large number of mutations were not detected (<60×) in TN4 and TN5. Similarly, the downsampling of the coverage led to fewer cells detected with less than 100× depth, which was problematic below 60× coverage depth (Figure S1G). Conversely, the average GI and allelic dropout of all samples showed little changes as the coverage decreased (Figures S1H and S1I). Finally, we investigated the ability to detect subclonal populations as the number of mutations and cells were downsampled (Figures S1J and S1K). These data showed that for the polyclonal samples, 38 mutations were required to detect all 4 subclusters in TN4 (15 mutations for 3 clusters), while 14 mutations were needed to detect all 3 subclusters in TN2. In contrast in the monoclonal tumors, all samples were able to detect 2 subclusters (normal and tumor) for lower numbers of mutations (>3 mutations). We then downsampled the number of cells, which showed that 1,350 cells were needed to detect 4 clusters for TN4 (280 cells for 3 clusters) and that 300 cells were needed to detect 3 clusters for TN2 (>50 cells to determine 2 clusters in all samples). Overall, these data suggest that a minimum of 38 mutations and 1,350 cells were required to resolve the clonal substructure in the polyclonal TNBC tumors.

## Discussion

Here, we report a new approach (MPT) that involves first performing deep-exome sequencing across a cohort of patients (e.g., 5–10) and identifying mutations that can be pooled together to develop a custom capture platform that targets only these genomic regions for sequencing in scDNA-seq experiments. Our study utilized a microdroplet-based scDNA-seq platform (Mission Bio) to perform high-throughput analysis of thousands of cells in parallel from 5 patients with TNBC. By targeting regions with somatic mutations (identified *a priori* by bulk exome sequencing), we were able to profile 330 mutations in thousands of single cells using MPT. In contrast, previous studies that have used the same microdroplet platform (Mission Bio) to profile patients with acute myeloid leukemia (AML) with pre-defined tumor panels typically only measure a very small set of mutations (∼1–8) in each patient across many cells.^27^ From these data, we obtained sufficient genomic markers in each patient to resolve clonal substructure, reconstruct clonal lineages, and identify putative driver mutations that have a potential functional impact during breast tumor progression.

Our study showed that 3 tumors were monoclonal (TN1, TN3, TN5), while 2 tumors (TN2, TN4) were multi-clonal, out of 5 patients analyzed. The samples showed a high degree of interpatient heterogeneity, in which single cells were well separated by patient clusters in high-dimensional space (Figure S3). We detected *TP53* mutations in all 5 patients with TNBC, which we inferred to be one of the earliest truncal mutations that was acquired during tumor evolution. Additionally, our FACS data show that genome doubling occurred in all 5 patients with TNBC as evidenced by increased DNA ploidy (>2.65N), which is consistent with other studies in TNBC.^11^ However, in addition to *TP53*, several additional early mutations and CNAs were also detected in the two polyclonal tumors. In TN4, early truncal homozygous mutations (*GRIN3A*, *PTPN4*, *AKAP6*) along with copy-number losses (*TP53*, *NOTCH3*) and gains (DMKN, *GRIN3A*) co-occurred with *TP53* in the MRCA at the earliest stages of tumor progression. In TN2, early truncal homozygous mutations (*ZMAT1*, *SULTIE1*) and copy-number losses (*TP53*, *ZMAT1*, *COL5A3*) and gains (*USP21*, *TMTC1*, *ZFHX4*[G/A]) co-occurred with *TP53* mutations in the MRCA at the earliest stage of progression. These data are consistent with a PCNE model in which short bursts of genome instability give rise to multiple clones that stably expand to form the tumor mass, as previously reported in TNBC.^5^^,^^11^ However, our data further suggest the possibility that mutations may also co-occur in short bursts of evolution, followed by stable clonal expansions that form the tumor mass in patients with TNBC.

Our findings on very limited numbers of gradual intermediate mutations occurring between the lineages of subclones in the polyclonal tumors and that there are few mutations occurring outside of the dominant clones in the monoclonal tumors is unexpected. While TNBC tumors were shown to evolve through PCNE, resulting in a limited number of subclones with highly clonal CNAs, the diversity of somatic point mutations has not been investigated widely through scDNA-seq methods.^5^^,^^11^^,^^16^ Previous bulk DNA-seq studies of TNBC tumors have reported high mutation burden, but they have not reported mutation diversity with high granularity beyond clustering of the bulk VAFs.^4^^,^^7^ Our study shows that the TNBC tumors profiled with MPT showed limited clonal diversity, consistent with the scDNA-seq copy-number studies.^5^^,^^11^^,^^16^ These data suggest that either (1) the mutations were acquired simultaneously in short bursts of evolution and the mutation rate is very low outside of these events or (2) there are large selective sweeps leading to dominant clones that outcompete the other clones that harbored the gradual intermediate mutations. From our current data, which are derived from a single time point, we cannot accurately distinguish between these two scenarios. Additionally, MPT may be potentially underestimating subclonal diversity in tumor samples that harbor few exonic mutations (N < 14) based on our downsampling calculations, making it more challenging to delineate intratumor heterogeneity and clonality in these tumors (Figures S1J and S1K). Another possible limitation is that the MPT approach focused on profiling only targeted mutation sites in exons that were identified by bulk exome sequencing and did not perform unbiased profiling of mutation sites across the whole genome (e.g., single-cell exome sequencing or WGS), potentially missing gradual intermediate mutations that may have occurred in single cells in non-coding and intergenic regions. While we primarily focused on exonic mutations in this study, due to the limited size of the custom amplicon panel (∼380 amplicons), future studies may include different classes of mutations (e.g., intronic, intergenic, insertions or deletions [indels]). This will become more feasible with future technical advances on expanding the Mission Bio targeted panels.^29^^,^^31^

To better understand the concordance of MPT to bulk exome sequencing or WGS, we directly compared these independent datasets from the same tumors in 5 patients. Comparison of VAFs between the bulk and MPT-merged single-cell data showed a very high concordance (average [avg.] Pearson correlation: 0.876), confirming the accuracy of the MPT. Copy-number estimation was performed on consensus clusters of the single-cell MPT data for each mutation and compared with a pseudo-bulk-derived reference. Even though the MPT estimation was limited due to coverage of only one amplicon per SNV, the copy-number estimations showed a high concordance as well (avg. Pearson correlation: 0.871) when performed on groups of cells from each subclone cluster. These data allowed us to obtain both mutation information and copy-number states for each subclone in the TNBC tumors for reconstructing their order in the clonal lineages during tumor evolution. Our data also suggest that PyClone2 generally overclustered the bulk exome data and estimated a higher number of subclones in each tumor compared with the scDNA-seq experiments, where Pyclone2 has been shown to have difficulty in assessing single-sample clonal structure with small numbers of clones (2–3 clusters).^34^ In our data, the bulk-exome approach (DNA-seq) alone was unable to provide an accurate estimate of clonal substructure of the tumors due to the inability of correctly determining the co-existence of mutations in each subclone. This analysis highlights the importance of using single-cell sequencing approaches to accurately resolve clonal substructure in solid tumors.

Important future directions will include applying MPT to study early-stage breast cancers such as atypical ductal hyperplasia (ADH) or DCIS to better understand mutation bursts and early events that initiate breast cancers in larger cohorts of patients. Additionally, it will be important to link clinical data on drug responses, survival, and progression to the amount of intratumor heterogeneity in invasive breast cancers and other cancer types. Overall, the MPT approach provides a powerful new tool to target only information-rich genomic regions for scDNA-seq analysis to study clonal substructure and lineages across diverse solid cancer types.

### Limitations of this study

This study has several notable limitations. First, since this project was primarily for the development of the MPT approach, only 5 patients with TNBC were analyzed. Thus, our biological conclusions regarding the evolutionary history of the tumors may not be generalizable across all TNBC cancers. Additionally, the custom amplicon panel included a limited number of targeted mutation sites (N = 330); however, future developments of the microdroplet technology (Mission Bio) will likely expand the number of amplicons to thousands of sites in the future. Moreover, while starting with mutations detected by bulk sequencing effectively limits the sequencing space in a cost-effective manner, WGS could potentially detect a greater number of mutations, due to increased sensitivity, providing a higher resolution of clonal diversity. Another limitation was that the copy-number measurements were not based on whole-genome measurements but were instead restricted to the targeted genomic regions covered by the mutations, thus limiting the overall genomic resolution of detection. While the MPT Mission Bio copy-number estimations generally showed high correlation to the pseudo-bulk reference, they are not as accurate as the copy numbers profiled using WGS scDNA-seq methods, which amplify DNA evenly across the whole genome. A further limitation is that was while indels and intronic and intergenic regions were not profiled in this study, these events could potentially be used to further resolve ITH and clonal diversity, as other studies have shown.^29^^,^^31^ Finally, it is important to note that the bulk and single-cell data were acquired from different spatial regions in the tumors, respectively, which could potentially impact the conclusions regarding clonality.

## STAR★Methods

### Key resources table

**Table:** 

REAGENT or RESOURCE	SOURCE	IDENTIFIER
**Biological samples**		

DCIS/IDC Breast Tumor Samples	University of Texas MD Anderson Cancer Center Breast Cancer Tissue Bank	https://www.mdanderson.org/research/research-resources/core-facilities/institutional-tissue-bank.html

**Chemicals, peptides, and recombinant proteins**		

Tagmentation reagents (Tn5 transposase, ATM)	Illumina	Cat# FC-131-1096
Ampure XP Beads	Beckman Coulter	Cat# A63880

**Critical commercial assays**		

Zymo DNA Clean & Concentrator Column Kit	Zymo	Cat# D4004
2X HiFi HotStart Ready Mix	Roche	Cat# KK2602
NEBNext end repair	NEB	Cat# E6050L
Quick Ligation	NEB	Cat# E6056L
dA-tailing	NEB	Cat# E6053L
NEBNext HiFi2x PCRmix	NEB	Cat# M0541L
Nimblegen SeqCap EZ Exome V2 kit	Roche	Cat# 05860482001
Mission Bio Custom Panel	Mission Bio	Cat# 145936
Mission Bio Cartridge	Mission Bio	Cat# 046459
Mission Bio Reagent Kit	Mission Bio	Cat# 165919

**Deposited data**		

Human reference genome NCBI build 37, GRCh37	Genome Reference Consortium	http://www.ncbi.nlm.nih.gov/projects/genome/assembly/grc/human/
FASTQ Files Bulk Exome Sequencing	This paper	SRA: PRJNA763862
FASTQ Files Mission Bio Single Cell Sequencing	This paper	SRA: PRJNA763862
FASTQ Files Single Cell Copy Number Sequencing	Minussi	SRA: PRJNA629885

**Oligonucleotides**		

5′-CAAGCAGAAGACGGCATACGAGA TXXXXXXXXGTCTCGTGGGCTCGG-3′	Illumina	Cat# FC-131-1096
5′-AATGATACGGCGACCACCG’AGAT CTACACXXXXXXXXTCGTCGGCAGCGTC-3′	Illumina	Cat# FC-131-1096

**Software and algorithms**		

Bowtie2(v2.2.6)	Langmead et al., 2012^35^	https://github.com/BenLangmead/bowtie2
SAMtools(v1.2)	Li et al., 2009^36^	https://github.com/samtools/; RRID: SCR_002105
DNACopy(v1.70)	Seshan et al., 2022^37^	https://bioconductor.org/packages/release/bioc/html/DNAcopy.html; RRID:SCR_012560
Dbscan(v1.1-5)	Hahsler et al., 2019^38^	https://github.com/mhahsler/dbscan
Picard(2.27.4)	Broad Institute, 2019^39^	https://broadinstitute.github.io/picard; RRID: SCR_006525
BEDTools(v2.28)	Quinlan et al., 2010^40^	https://github.com/arq5x/bedtools2; RRID: SCR_006646
GATK(4.1.9)	McKenna et al., 2010^41^	https://github.com/broadinstitute/gatk/releases; RRID: SCR_001876
ANNOVAR(v2019Oct24)	Wang et al., 2010^42^	https://github.com/WGLab/doc-ANNOVAR
MutSignatures(v2.1.1)	Fantini et al., 2020^43^	https://github.com/dami82/mutSignatures
Cutadapt(v3.1)	Martin et al., 2011^44^	https://github.com/marcelm/cutadapt; RRID: SCR_011841
BWA-MEM(v0.7.17)	Langmead et al., 2009^45^	https://github.com/lh3/bwa; RRID: SCR_022192
Mission Bio Tapestri Insights(v2.0)	Mission Bio, 2020^46^	https://portal.missionbio.com/
UMAP(v0.2.4)	McInnes et al., 2018^47^	https://github.com/tkonopka/umap; RRID: SCR_018217
stats(v3.6.2)	R Core Team, 2013^48^	https://www.r-project.org
ComplexHeatMap(v2.2.0)	Gu et al., 2016^49^	https://github.com/jokergoo/ComplexHeatmap
PolyPhen(v2.2.3)	Adzhubei et al., 2010^50^	http://genetics.bwh.harvard.edu/pph2/
SIFT(6.2.1)	Vaser et al., 2016^51^	https://sift.bii.a-star.edu.sg; RRID:SCR_012813
CADD(v1.4)	Kircher et al., 2014^32^	https://cadd.gs.washington.edu/; RRID:SCR_018393
Ape(v5.3)	Paradis et al., 2004^52^	https://github.com/craN/Ape; RRID:SCR_017343
Tapestri.cnv(v1.0)	MissionBio, 2020^53^	https://portal.missionbio.com/
Pyclone2(v2.91)	Roth et al., 2014^33^	https://github.com/Roth-Lab/pyclone; RRID:SCR_016873
Gini(v0.1.0)	Brown et al., 1994^54^	https://github.com/PABalland/EconGeo/blob/master/R/Gini.r
Seqtk(v1.3)	Li, 2013^55^	https://github.com/lh3/seqtk; RRID:SCR_018927

**Other**		

FACS Aria II	BD Biosciences	https://www.bdbiosciences.com/en-us/products/instruments/flow-cytometers/research-cell-sorters/bd-facsaria-iii
BD FACSMelody	BD Biosciences	https://www.bdbiosciences.com/en-us/products/instruments/flow-cytometers/research-cell-sorters/bd-facsmelody
Echo 525	Labcyte	https://www.beckman.com/liquid-handlers/echo-525
HiSeq 4000	Illumina	https://www.illumina.com/systems/sequencing-platforms/hiseq-3000-4000.html
Mission Bio Tapestri	Mission Bio	https://missionbio.com/products/platform/
TapeStation	Agilent	https://www.agilent.com/en/product/automated-electrophoresis/tapestation-systems/tapestation-instruments/4200-tapestation-system-228263

### Resource availability

#### Lead contact

Further information and requests should be directed to the lead contact, Nicholas E. Navin (nnavin@mdanderson.org).

#### Materials availability

This study did not generate new unique reagents.

### Experimental model and subject details

#### Triple negative breast cancer tumor samples

We selected five snap-frozen tissue samples from women with untreated intraductal carcinoma breast cancer, including paired normal adjacent breast tissues, that were collected from the University of Texas MD Anderson Cancer Center Breast Tissue Bank. The patients were chosen based on their tumor grade (stage 3), age (37–71), histopathology, lack of treatment, tumor ploidy, and triple-negative (ER, PR, Her2) receptor status (Table S1). IHC confirmed the ER and PR status of each tumor to be <1%, while Her2 negative status was determined by IHC or FISH cytogenetic analysis. All of the tissues were collected under informed consent, and the IRB for this study was approved by the MD Anderson Cancer Center Review Board.

### Method details

#### Generation of nuclear suspensions from frozen tissues

For each bulk (tumor and normal) and single cell experiment, nuclear suspensions were generated from frozen tissue using an NST/DAPI buffer (800 mL of NST [146 mM NaCl, 10 mM Tris base at pH 7.8, 1 mM CaCl_2_, 0.05% BSA, 0.2% Nonidet P-40, and 21 mM MgCl_2_]), 200 mL of 106 mM MgCl_2_ and 10 mg DAPI.^16^ To prepare the nuclear suspensions, sections of the tumors were minced with a surgical blade in petri dishes containing NST/DAPI, and then nuclei were filtered through a 40 um mesh into 1.5 mL Eppendorf tubes. Cell counts were obtained from DAPI utilizing the Countess (Invitrogen).

#### FACS enrichment of aneuploid nuclei

In order to enrich tumor nuclei from the tissue sections, millions of DAPI-stained nuclei were flow-sorted on the FACS Aria II (BD Biosciences). Nuclei were selected from the aneuploid distributions based on their DAPI intensity (>2N) relative to the diploid peaks of nuclei (2N) and deposited in 1.5 ML Eppendorf tubes. Notably, the FACS enriched bulk aneuploid tumor nuclei suspensions were used for downstream bulk exome and pseudo-bulk copy number profiling, while the scDNA Mission Bio experiments were all conducted with unsorted cells to provide for copy number estimation (that were not enriched by FACS).

#### Pseudo-bulk copy number sequencing

Low pass single cell whole genome sequencing was performed on the FACS enriched aneuploid nuclear suspensions of the tumor samples following the Acoustic Tagmentation protocol (ACT). The FACS sorted aneuploid nuclei were distributed into 384-well plates, where the Echo525 platform (Labcyte) was utilized to dispense lysis buffer [Protease (1.36AU/mL) diluted 1:9 in 5% Tween 20, 0.5% Triton X-100 and 30mM Tris pH 8.0], tagmentation reagents ((TD:ATM 2:1, 384PP-Plus_GPSA), and barcoding reagents [(5′-CAAGCAGAAGACGGCATACGAGAT**XXXXXXXX**GTCTCGTGGGCTCGG-3′) and S5XX (5′-AATGATACGGCGACCACCGAGATCTACAC**XXXXXXXX**TCGTCGGCAGCGTC-3′) primers (384PP_AQBP)] in 2X HiFi HotStart Ready Mix (6RES_GPSA) in subsequent controlled steps at the nano-liter scale. The final libraries were purified by 1.8X ampure XP beads and sequenced at 76-single read cycles on the Illumina HiSeq 4000 sequencer. A comprehensive outline of each specific step of the ACT method can be found in a previous publication.^11^

#### Pseudo-bulk copy number detection pipeline

The resulting sequencing reads were demultiplexed into single cell FASTQ files and aligned to h19 using bowtie2 and converted form SAM to BAM files using SAMtools.^35^^,^^36^ Aligned reads were counted in variable bins (220kb), normalized for GC content, bin-wide ratios were calculated, and segmentation was applied with circular binary segmentation (CBS). Segments were merged to attach adjacent segments by R Bioconductor DNACopy package and outliers were removed by dbscan.^37^^,^^38^ Integer copy number was calculated from the DAPI signal in the FACS data for each tumor sample, where the first “D” peak is considered to be reference (2N) and the ratio of the “A” peak to the “D” peak gives the average ploidy. The inferred segment ratios in each sample are then multiplied by the FACS derived ploidy to estimate the final integer copy number. Finally, the consensus (pseudo-bulk) copy number of each sample was calculated by taking median of the *i*th segment of all single cell inferred integer copy number profiles for each respective sample. The full copy number pipeline and analysis (including scripts) can be found in more detail in a previous publication.^11^

#### Bulk exome sequencing

After using FACS to enrich aneuploid nuclear suspensions or matched normal tissues, the nuclei were prepared for sequencing by first fragmenting the DNA to 250 bp using a Covaris Sonicater and subsequently purifying with the Zymo DNA Clean & Concentrator Column kit (Zymo). Next, the fragmented DNA was barcoded using the NEBNext end repair model (NEB), dA-tailing (NEB), and quick ligation (NEB); and then library amplification was performed by PCR using NEBNext HiFi2x PCRmix. An exome capture was then applied using the Nimblegen SeqCap EZ Exome V2 kit (Roche), and both normal/tumor matched samples were sequenced paired-end at 100 bp on the Illumina HiSeq4000.

#### Detection of mutations in bulk DNA samples

The resulting FASTQ files from the Hiseq4000 sequencing run of normal/tumor matched samples were then demultiplexed using our custom software code (deplexer.pl). Bowtie 2 was then applied to align each FASTQ to the human genome reference (hg19) and then converted to individual BAM files by SAMtools.^35^^,^^36^ Picard was used to remove PCR duplicates from the resulting BAM files.^39^ Indel regions were then realigned using the Genome Analysis Toolkit (GATK), and sequencing reads were filtered at a standard mapping quality score of 40.^41^ BEDTools was utilized to calculate coverage depth and breadth.^40^ The full in-house pipeline and scripts outlining each step can be downloaded from *Nature Protocols*, and the full bulk sequencing metrics can be found in (Table S2).^56^ GATK was then applied with default parameters to detect variants and recalibrate quality scores resulting in a final bulk VCF file summarizing the results. Mutations were filtered out based on two distinct criteria: consensus filtering (mutations must be detected in at least 3 reads) and clustered regions (multiple mutations detected in a 10-bp sliding window). The resulting variants were then annotated by applying ANNOVAR to the VCF files using default parameters.^16^^,^^42^ Sites having low coverage (<10X) were annotated as missing values (NA) and nonvariant sites were labeled as germline reference. Classes of exonic mutations were annotated by Annovar and mutational signature analysis was performed using the MutSignatures R package.^43^

#### Mission Bio general custom panel

Mission Bio’s custom design targeted panels were used to choose single nucleotide variants to be profiled over specific amplicon regions for high-throughput single cell DNA sequencing. Amplicon-based targeted sequencing was then applied to profile SNVs and estimate copy number variation (CNV) variation in up to 10,000 cells per sample. Mission Bio custom panels generally consist of short amplicons ranging in size from 175 to 275 bp with primers of 18–35 bp in length. The original custom panels that we utilized contained up to 380 amplicons, while future custom panels are expected that have 1,000 or more amplicons. Although most genes can successfully be profiled by the custom panels, several technical limitations do exist that can exclude some gene target areas from the final panel. Most targets are missed because of the GC%, where only genes in regions with 27–70% GC content and primers with 27–62% GC can be included in the panel. Amplicons also must be non-overlapping to be included; however, amplicons can be tiled end-to-end to address this issue, where tiling can also be employed to handle genes longer that 250 bp (>250bp can have center gap regions with low coverage). Mission Bio custom panels also cannot contain genes located in masked regions of the genome (eg. centromere regions, telomere regions). Highly repetitive regions (eg. CAG repeat) or regions with high homology (eg. LINEs, SINEs, homologous genes) are very difficult to profile, and require advanced approaches (250 PE sequencing, custom reference genome) on a case by case basis.

#### Mission Bio custom MPT panel

In order to capture the most variance and heterogeneity in our tumor samples, we selected variants with allele frequencies ranging from 0.1% to 100% to identify the heterogeneous subclonal events to build the MPT panel. Out of the 379 genes we submitted for the custom MPT panel, 330(87%) genes (TN1 - 92%, TN2 - 89%, TN3 - 82%, TN4 - 85%, TN5 - 87%) were selected to create the panel, where the designed amplicon range was 125–190bp (43 amplicons ≤ 140bp, 275 at 140–175bp, 12 amplicons >175bp). The majority of the amplicons were excluded from our panel due to high GC% (>70%), overlapping amplicons, and amplicon length (>275 bp). Notably, 9 low frequency mutations (MED24, BICD1, TBX4, OTUD5, PRDM5, SENP2, MARCH6, PODXL2, ADCY8) in TN3 and TN5 (9/330) are possible FPs since they were found in a relatively low frequency in a small number of cells, however, they were retained in the custom panel due to a high concordance of allele frequencies with the bulk data. The final custom panel of 330 amplicons was then used on the Mission Bio Tapestri platform to profile SNVs utilizing targeted high-throughput DNA sequencing.

#### Mission Bio single cell DNA sequencing

The MPT sequencing method utilizes the Mission Bio Tapestri microdroplet platform to perform targeted high-throughput single cell DNA sequencing, where we followed the basic Mission Bio Tapestri default guidelines (Mission Bio User Guide). After isolating single cells in NST/DAPI buffer from the frozen tissue, we input 2–4,000 cells/uL into the Mission Bio Tapestri cartridge, where single cells are individually partitioned into nano-droplets with lysis buffer (Mission Bio) and incubated at 50C for 60 min on the PCR block (Bio Rad) to release the DNA. The DNA is then re-loaded into the Mission Bio cartridge, where barcoding beads and PCR reagents are combined in a second merged encapsulation. UV light (Analytik Jena XX-15L) is applied for 8 min to release the barcoded DNA from the beads, and the DNA is amplified via multiplexed PCR (Bio Rad) within the droplets (Mission Bio). The droplet emulsions are then broken and the DNA is extracted and purified using Ampure XP Beads (0.72X). Qubit Fluorescence Quantification (Invitrogen) was used the quantify the concentration of DNA at an expected range of (0.2–4 ng/uL). For library construction, the i5 and i7 indexes are added and library amplification is performed (Mission Bio) on the PCR block (Bio Rad). The library is then purified using Ampure XP beads (0.69X) for an expected on-target size range of (350–550 bp) at a range of concentration (2–20 ng/uL). Qubit is used as a preliminary raw quantification of DNA concentration followed by the TapeStation (Agilent) for a more detailed view of the fragment size distribution in addition to the targeted concentration. The library was then diluted to 5nM (0.9–1.3 ng/uL) and sequenced on the Illumina HiSeq4000 at 150 paired-end.

### Quantification and statistical analysis

#### Mission Bio mutation detection **pipeline**

The resulting single cell demultiplexed FASTQ files were input into the Tapestri Sequencing Pipeline (Mission Bio). Adapter sequences are first trimmed from the raw sequencing reads using Cutadapt.^44^^,^^57^ Short reads less than 30 nt are discarded and barcodes are extracted from the reads. Error correction is used (Hamming distance or Levenshtein distance) to correct barcodes with a partial match to increase yields. A cell calling algorithm is then applied to only select cells that have at least 80% amplicon read completeness and pass a total reads cutoff. The reads are then mapped to the reference genome (hg19) using the BWA-MEM algorithm with default parameters discarding any unmapped reads.^45^^,^^58^ The cells are genotyped using GATK with a joint calling approach that follows GATK Best Practices.^59^^,^^60^ Finally, joint genotyping is performed for all cells using GATK’s GenotypeGVCFs tool, VCF and HDF5 files are generated, and the genotypes and cell matrix are converted into an open-source loom format for further analysis.^61^ Full MPT single cell sequencing metrics can be found in (Table S3). Loom files were input into Mission Bio Tapestri Insights, where default parameters were used to filter the data by cell quality (<30), read depth (<10), alternate allele frequency (<20%), variants genotyped/cell (<50%), percent genotypes present (<50%) and percent cells mutated (<1%).^46^ Heterozygous germline mutations present in more than 95% of cells were also removed, and amplicons and/or cells that had NA values in over 50% of cells were excluded. Multiple mutations occurring in the same gene in one patient are indicated by a base pair change annotation next to the mutation gene name. For example, XIRP1[C/T] refers to a mutation with a base pair change of C- > T that has occurred in this gene.

#### Clustering

To investigate the clonality of the tumor samples, a multi-step clustering process was applied to the remaining cells, where PCA (prcomp()) was initially employed to reduce the high dimensional space.^48^^,^^62^ UMAP (umap(), n_neighbors = 15, a = 1, b = 1) was further applied to the PCA selected features to partition the cells in distinct clusters where additional outliers (eg. low quality, depth, NAs) were further excluded.^47^ Technical noise (over-sensitivity) is observed as excess dispersion in the UMAP clustering due to the highly clonal nature of the samples (Figures 3, 4 and Table S3). Unsupervised hierarchical clustering via ComplexHeatMap (Heatmap()) was next applied to the distinct clusters to provide annotated single cell granularity per cluster across all amplicons and visualize the final subclones based on the determined features (linkage = complete, distance = Euclidean), which were then overlaid back on the UMAP projection for annotation (Figures 3, 4 and Table S3).^49^ Inter-patient heterogeneity was characterized by combining the tumor cells and mutations from all 5 patients, and performing clustering with UMAP (n = 13,200, umap(), n_neighbors = 15, a = 1.2, b = 1.2) to define clusters (excluding outliers as above) for each tumor sample (Figure S3).^47^

#### Estimation of mutation impact

To predict the functional impact of the detected variants, Combined Annotation-Dependent Depletion (CADD) was coupled with PolyPhen2 and SIFT to prioritize causal variants based on over 60 combined genomic features.^32^^,^^50^^,^^51^ Most annotations tend to exploit a single information type and/or are restricted in scope, so a broadly applicable metric that objectively weights and integrates diverse information was needed. Thus, CADD integrates multiple annotations into one metric by contrasting variants that survived natural selection with simulated mutations. For each sample, the mutations were first filtered by a PolyPhen or (1-SIFT) score over 0.8, then the top 30 deleterious genes (highest potential functional impact) were selected from this filtered pool based on the CADD functional impact scores for each patient scaled by CADD >10 = top 10%, CADD >20 = top 1%, and CADD >30 = top 0.1% (Figures 3 and 4).

#### Integrated phylogenic tree

Neighbor Joining (NJ) trees were constructed from the clustered SNV data using Ape (dist(), nj(), Table S3), and the cluster annotations defined through UMAP and hierarchical clustering were overlaid onto the resulting NJ trees.^52^ The respective mutations for each sample were annotated on the NJ tree based on the clusters in which they were identified. Estimated copy number aberrations for deleterious genes showing significant CNV per cluster were annotated on the trees based on the clusters in which they were identified (Figures 3 and 4).

#### Mission Bio copy number estimation

Tapestri.cnv is an R package provided by Mission Bio for estimating copy number from single cell sequencing read depth.^53^ A higher degree of variance is commonly observed in read depth coverage with amplicon-based sequencing, which can be observed in the calculated GI and CV across all cells and amplicons (Figures S1A and S1B). This can potentially confound copy number estimation, where some amplicons or cells can have a significantly higher or lower coverage depth in comparison making normalization imperative. By performing a normalization of the read count matrix for each tumor sample, we can mitigate the effects of this non-uniformity in regard to the coverage depth, thus allowing for a more accurate copy number inference. To normalize for cells, the read depth for each mutation in a respective cell is divided by the total number of reads for the entire cell across all amplicons. To both normalize for amplicon read depth and estimate the copy number of each cell, we leverage the relationship between each mutation’s tumor profile and its associated normal (reference) population. Specifically, the median coverage read depth was first calculated for all normal (reference) single cells in each respective amplicon. Next, each tumor cell in a specific amplicon is then divided by the median of its respective normal (reference) population (eg. the normalized read count for TP53 mutation in each tumor cell in sample TN4 is divided by the median of the normalized read count for all TN4 TP53 normal (reference) cells). This both normalizes the amplicon read depth to attenuate for the high variance between each amplicon and effectively imparts a normalized copy number ratio value for each cell per each specific mutation. This effective copy ratio value then serves as the estimated copy number, which is simply the normalized read count of each tumor cell mutation divided by its respective median normal reference. While this provides single cell copy number estimations per cell, the data has significant technical noise at single cell resolution. To correct for this, we calculated the median estimated copy number for each mutation per cluster, which was used to annotate hierarchal clustering heat maps and integrate NJ trees (Figures 3 and 4). To compare the accuracy of the single cell copy number estimations from the MPT panel, whole-genome sparse depth sequencing was performed on each sample, which served as a pseudo-bulk gold standard comparison (Figure 2B).

#### Single cell vs bulk exome comparisons

The median copy number ratio values estimated from the Mission Bio data per gene were compared against a ‘gold standard reference’ that was computed by combining consensus single cell copy number data (TN1 - 1,378, TN2 - 1,224, TN3 - 1,307, TN4 - 1,101, TN5 - 1,238) from each tumor to generate a pseudo-bulk profile (Figure 2B). Correlation was calculated (cor.test()) for all filtered mutations using Pearson (t = t-statistic, df = degrees of freedom(df = (n-1)(k-1); k = 2), CI = 95% confidence interval) [TN1 - t = 19.383, df = 15, CI = (0.7508439, 0.8171596), TN2 - t = 10.537, df = 18, CI = (0.8363862, 0.9707934), TN3 - t = 6.4446, df = 10, ci = (0.7260698, 0.9522813), TN4 - t = 6.0815, df = 68, CI = (0.6427014, 0.9731556), TN5 - t = 11.286, df = 46, CI = (0.8363862, 0.9207934)] (Figure 5A).^48^ Next, the allele frequencies of all variants in the Mission Bio and bulk data were compared, and the relationship was defined by Pearson’s correlation (cor.test()) [TN1 - t = 7.1999, df = 24, CI = (0.6758093, 0.9236741), TN2 - t = 11.971, df = 29, CI = (0.7887095, 0.9219351), TN3 - t = 18.8136, df = 17, CI = (0.8569058, 0.9207677), TN4 - t = 28.602, df = 74, CI = (0.8579727, 0.8957873), TN5 - t = 26.02, df = 54, CI = (0.8971459, 0.9383571)] (Figure 5B).^48^ Pyclone2 was applied to the bulk exome data using default parameters to estimate the total number and size of the clusters.^33^ The resulting Pyclone2 cluster assignments were then compared to the clusters defined by the single cell analysis (Figures 5C and S4).

#### Doublet identification

To identify doublets in the dataset, we leveraged the normalized coverage read count matrix created using tapestri.cnv, where we utilize the normalized copy number ratio values (normalized read depth) to determine outliers. Notably, we sought to determine outlier clusters with significantly higher average normalized ratio values (effectively higher normalized coverage depth) to mark and remove cells as doublets. Unsupervised hierarchical clustering via ComplexHeatMap (Heatmap()) was applied to the matrix of normalized ratio values for each tumor cell across all mutations to define cluster differences per sample.^49^ For each defined cluster, the log2 of the average ratio value of all cells in each respective cluster was calculated across all mutations per sample. Thus, each cluster was assigned a respective metric (log2(Ratio Values)) quantifying its average normalized ratio value for comparison. In each tumor sample, a single outlier cluster, defined as [Doublet] cluster, was observed that had a significantly higher average normalized ratio value (higher read depth) when compared to the values of the other defined tumor clusters. To quantify this difference, we applied a paired t-test (t.test(paired = TRUE)) to compare the distributions of the ratio values of the [Doublet] cluster to the [Tumor] cluster (distribution of remaining cluster average ratio values) in each sample (eg. TN1 outlier cluster [Doublet] vs combined average ratio values of remaining TN1 tumor clusters [Tumor]), which simply shows that clusters containing cells with significantly higher normalized ratio values (akin to higher read depth) are marked as doublets/outliers and subsequently removed from the downstream analysis (mod = mean of the difference) [TN1 - t = −5.3145, df = 24, mod = −0.2915397, CI = (−0.4047607, −0.1783187), TN2 - t = −3.3362, df = 29, mod = −0.1337342, CI = (−0.26956914, −0.04789932), TN3 - t = −2.9942, df = 17, mod = −0.167538, CI = (−0.28559127, −0.04948482), TN4 - t = −3.2603, df = 74, mod = −0.1641321, CI = (−0.26458814, −0.06367613), TN5 - t = −5.766, df = 54, mod = −0.325912, CI = (0.439234, −0.212590)] (Figures S1D and S5).^48^

#### MPT benchmarking metrics

In order to access the efficiency, sensitivity, and limitations of the MPT approach, we calculated a variety of benchmarking metrics and several downsampled conditions. Amplicon sequencing can have a relatively non-linear distribution of read depth, so we examined the coverage read depth distributions of each tumor sample to access the extent of this variance (Figure S2B). Summary Gini index (GI ) (gini()) and coefficient of variation (CV) (SD/mean) were calculated for both the cells and amplicons by taking the average of both metrics for each sample (average of all cells/amplicons per sample) (Figures S1A and S1B, Table S3).^54^ To obtain a higher resolution, we next calculated the GI of each respective amplicon per sample to better understand the individual contribution of each amplicon to the variance (Figure S2A). For additional summary metrics, we calculated the average allelic dropout per sample (based on heterozygous SNPs - Mission Bio Pipeline), the percentage of doublets per tumor sample (STAR Methods), and the percentage of successful amplicons per sample (amplicons with mean reads above 0.2 ∗ the mean reads per cell per amplicon - Mission Bio Pipeline) (Figures S1C–S1E).

Seqtk was employed to downsample the coverage depth of each sample through random sampling of the FASTQ file reads for each depth (100X->10X).^55^ After re-running the Mission Bio pipeline with each of the downsampled FASTQs, the data was re-processed with the same analysis pipeline to yield both the number of amplicons and cells, respectively, that could be successfully profiled by MPT at each coverage depth (Figures S1F and S1G). Summary GI and allelic dropout were also calculated for each downsampled coverage condition per sample (Figures S1H and S1I). Finally, to investigate the sensitivity of MPT to accurately determine subclusters, we downsampled the number of mutations and cells, respectively, at the original coverage depths of each sample. -Specifically, we successively random sampled lower numbers of mutations and cells for each tumor sample mutation matrix. For each downsampled condition, we then re-analyzed the data to determine the number of clusters that could be still be successfully delineated at each condition (Figures S1J and S1K).

## Data Availability

The data has been deposited to the NCBI Sequencing Read Archive (SRA) under the accession SRA: PRJNA763862, PRJNA629885. Code is available upon request.

## Supplemental information

Document S1. Figures S1–S5 and Tables S1–S3

Document S2. Article plus supplemental information
