# Supplementary material for: Reconstructing mutational lineages in breast cancer by multi-patient-targeted single-cell DNA sequencing
Source: Cell Genom. 2022 Nov 9;3(1):100215. doi: 10.1016/j.xgen.2022.100215 (PMC9903705; doi:10.1016/j.xgen.2022.100215)
Supplement: Document S1. Figures S1–S5 and Tables S1–S3 [file mmc1.pdf]

Cell Genomics, Volume *3*

Supplemental information

Reconstructing mutational lineages in breast cancer by multi-patient-targeted single-cell DNA sequencing

Jake Leighton, Min Hu, Emi Sei, Funda Meric-Bernstam, and Nicholas E. Navin

Reconstructing mutational lineages in breast cancer by multi-patient-targeted single cell DNA sequencing

Leighton et al.

Supplementary Materials

Contents

Supplementary Figure 1
MPT Panel Summary Metrics and Benchmarking ……………..…………….…………….Page 2

Supplementary Figure 2
Amplicon Coverage Depth Distributions and Variance……………..…………….................Page 3

Supplementary Figure 3
High-dimensional analysis of inter-patient heterogeneity……………..…………...............Page 4

Supplementary Figure 4
Pyclone2 Subclone Frequencies from Bulk Exome Data ………...…………….……………Page 5

Supplementary Figure 5
Exclusion of Single Cell Doublets using Coverage Depth Distributions ……………………Page 6

Supplementary Table 1
Clinical Information on the TNBC Patients …………………………………………………..Page 7

Supplementary Table 2
Bulk DNA Exome Sequencing Metrics …………………………………………………………Page 8

Supplementary Table 3
MPT Single Cell DNA Sequencing Metrics …………………….……………………….……...Page 9

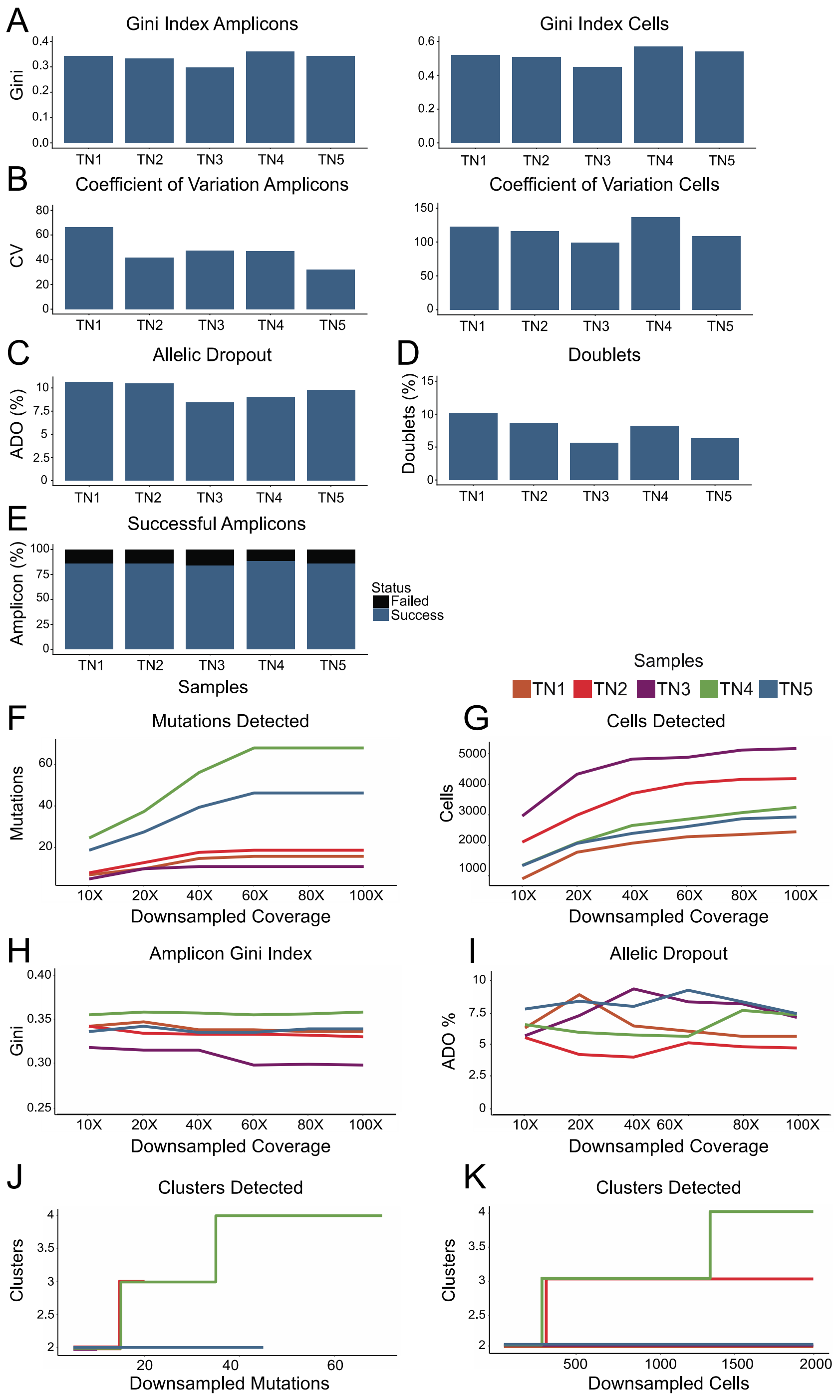

Supplementary Figure 1 - MPT Panel Summary Metrics and Benchmarking
(A) Average amplicon and cell Gini Indexes of coverage depth distributions. (B) Average amplicon and cell Coefficient of Variation of coverage depth distributions. (C) Average Allelic dropout from heterozygous SNPs. (D) Percentage of doublets identified in each of the tumor samples estimated from the doublet clusters. (E) Percentage of successful and failed amplicons. (F) Mutations detected after downsampling coverage depth. (G) Cells profiled after downsampling coverage depth. (H) Average amplicon Gini Index for downsampled coverage depth. (I) Allelic dropout estimated at downsampled coverage depth. (J) Subclone clusters detected after downsampling the number of mutations. (K) Subclone clusters detected after downsampling the number of cells. See methods sections for metric calculations (Average summary statistics and downsampling of cells/mutations used in Figure 3-5, Table S3, and STAR Methods).

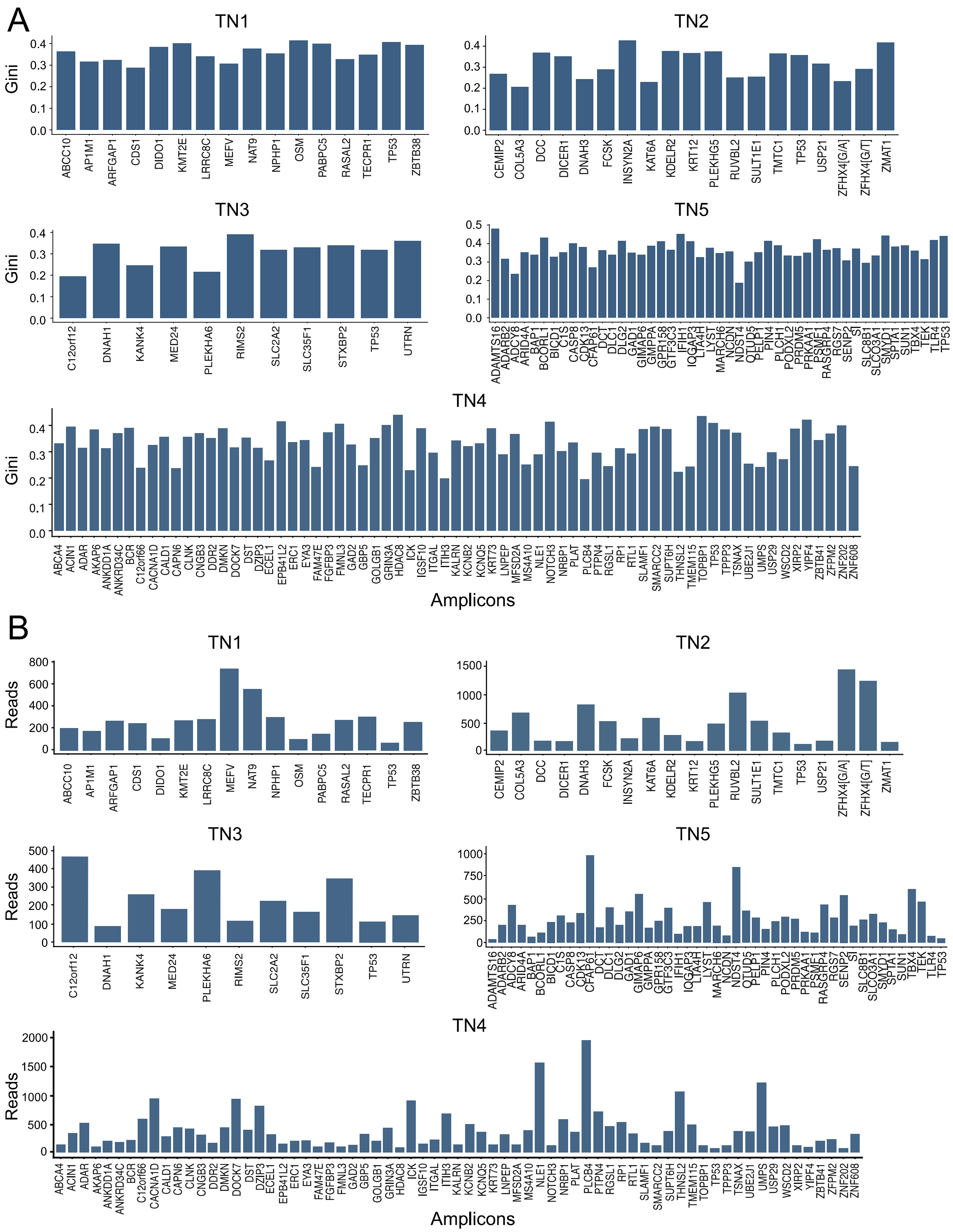

Supplementary Figure 2 - Amplicon Coverage Depth Distributions and Variance
(A) Gini Index calculated from coverage depth distributions of reads from each amplicon per tumor sample. (B) Coverage depth distribution of total reads for each amplicon for each tumor sample (Distribution and depth statistics of each mutation utilized in Figure 3-5, Table S3, and STAR Methods).

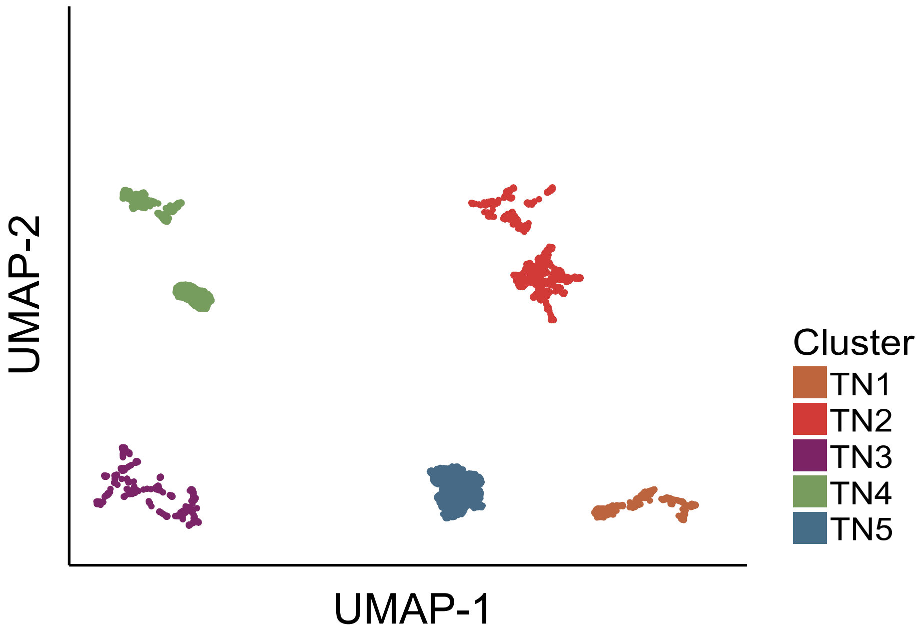

Supplementary Figure 3 - High-dimensional analysis of inter-patient heterogeneity
UMAP dimensional reduction of scDNA-seq mutation data from MPT for all 5 patients combined showing patient-specific clusters of cells (Discrete tumor populations of cells related to each sample in Figures 3-5, Table S3, and STAR Methods).

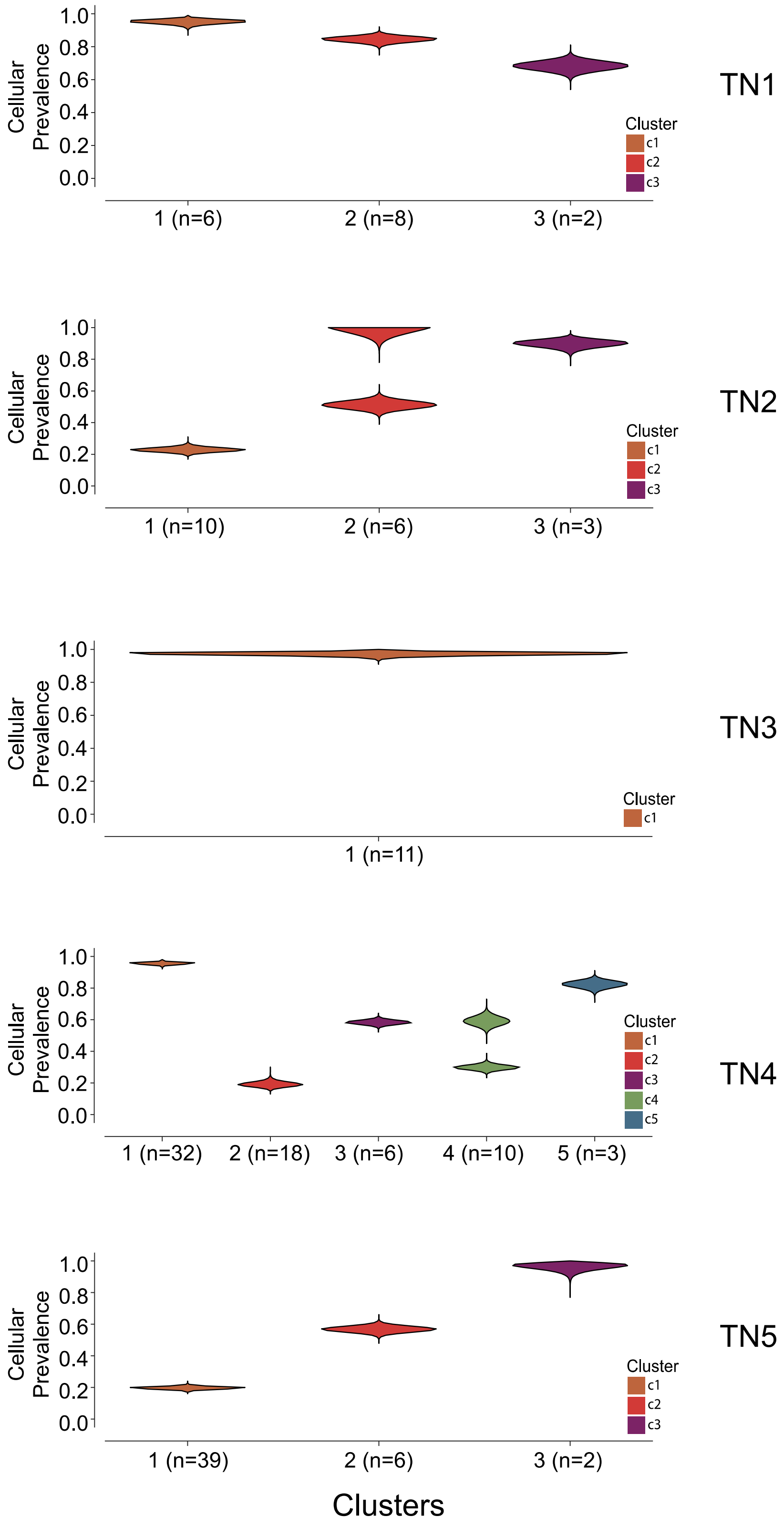

Supplementary Figure 4 - Pyclone2 Subclone Frequencies from Bulk Exome Data
Pyclone2 subclonal inference based on using the bulk exome mutation frequency data to estimate the size, number, and distribution of subclones in each of the 5 tumor samples (Distinct tumor populations of mutations utilized in Figure 3-5, Table S3, and STAR Methods).

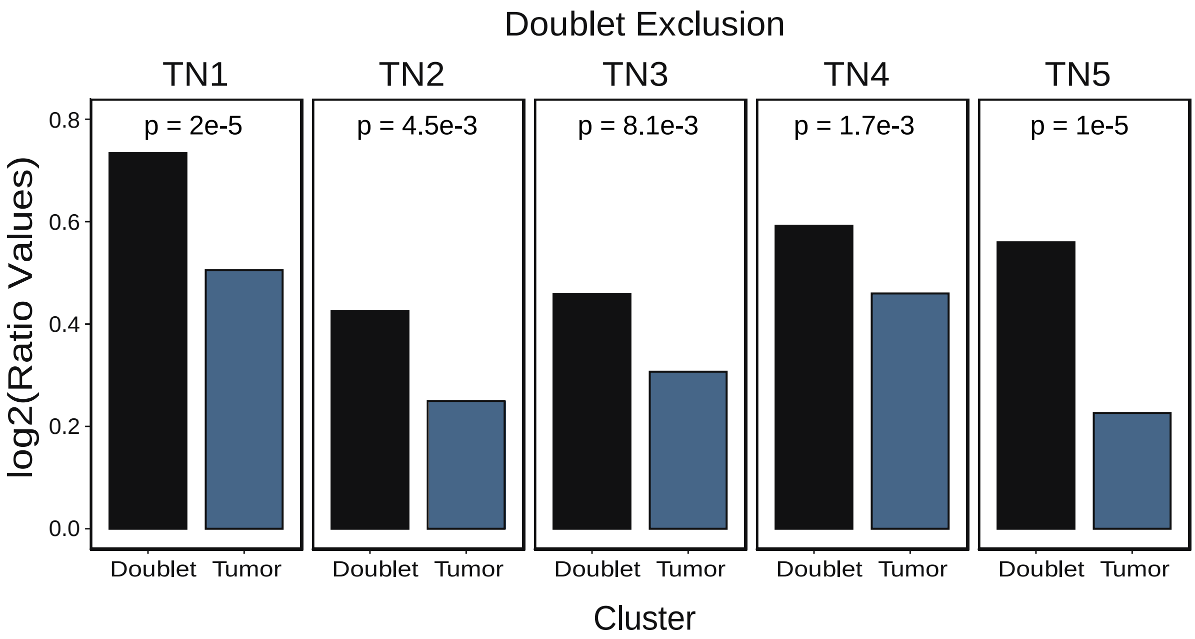

Supplementary Figure 5 - Exclusion of Single Cell Doublets using Coverage Depth Distributions

Single cell doublets clusters were identified and removed by comparison to all subclone cluster mutation depths. The coverage depths were normalized by using the log2 of the normalized average coverage depth read counts. Paired t-test was applied to determine significantly higher coverage depths for the doublet clusters (Excluded cell doublets in each sample related to Figure 3-4 and STAR Methods).

ID	Age	ER	PR	HER2	Grade	Pathology	Lymph	Treatment	Ploidy
TN1	50	<1%	<1%	neg	3	IDC	neg	untreated	3.1
TN2	56	<1%	<1%	neg	3	IDC/DCIS	neg	AG	3.7
TN3	53	<1%	<1%	neg	3	IDC/DCIS	neg	untreated	3.6
TN4	71	<1%	<1%	neg	3	IDC/DCIS	neg	untreated	3.4
TN5	37	<1%	<1%	neg	3	IDC/DCIS	neg	untreated	2.8

Supplementary Table 1 - Clinical Information on the TNBC Patients
Clinical information for the frozen tumor tissue specimens collected from five triple negative breast cancer patients at MD Anderson Cancer Center. Treatment included AG (Anastrozole, Gefitinib) in patient TN2, and the pathologies are defined as invasive ductal carcinoma (IDC) or ductal carcinoma in situ (DCIS). Ploidy was determined by FACS sorting of DAPI-stained nuclei relative to a normal 2N control (IDC/DCIS samples used for MPT approach in Figure 1 and STAR Methods).

Sample	Panel	Mapped Reads	Average Depth	PCR Duplication Rate	Coverage Breadth
TN1	Roche Exome Capture V2	145,527,833	145X	17.90%	99.80%
TN2	Roche Exome Capture V2	104,246,353	108X	15.60%	99.70%
TN3	Roche Exome Capture V2	90,979,658	91X	15.80%	99.50%
TN4	Roche Exome Capture V2	90,873,723	86X	14.10%	99.70%
TN5	Roche Exome Capture V2	100,059,432	106X	17.10%	99.70%

Supplementary Table 2 - Bulk DNA Exome Sequencing Metrics
Bulk DNA exome sequencing metrics from the five TNBC patients that were used to generate the MPT mutation panels (Bulk sequencing metrics related to samples profiled in Figure 1-2 and STAR Methods).

Sample	Panel	Cell Number	Average Depth	Panel Uniformity	Duplication Rate	Encapsulation Rate
TN1	Custom MPT	4,270	176X	86.13%	10.23%	4.00%
TN2	Custom MPT	4,141	160X	83.58%	9.05%	4.23%
TN3	Custom MPT	5,941	136X	88.32%	6.43%	5.30%
TN4	Custom MPT	5,172	175X	86.31%	8.62%	4.92%
TN5	Custom MPT	4,002	174X	86.16%	12.16%	5.71%

Supplementary Table 3 - MPT Single Cell DNA Sequencing Metrics
Single cell DNA sequencing metrics calculated from running the Mission Bio (Tapestri) microdroplet system with the Custom MPT panel for the 5 TNBC patients. Panel coverage uniformity, duplication rates and encapsulation rates were estimated from the Mission Bio Tapestri Insights Data processing pipelines (Single cell sequencing metrics related to samples profiled in Figure 1, 3-5 and STAR Methods).
